# Investigating the biology of yeast aging by single-cell RNA-seq

**DOI:** 10.18632/aging.204991

**Published:** 2023-08-14

**Authors:** Yi Zhang, Xiannian Zhang, Brian K. Kennedy

**Affiliations:** 1 Department of Strategy and Development, Sansure Biotech Inc., Changsha, Hunan 410205, China; 2 School of Basic Medical Sciences, Beijing Advanced Innovation Center for Human Brain Protection, Capital Medical University, Beijing, China; 3 Healthy Longevity Translational Research Programme, Yong Loo Lin School of Medicine, National University Singapore, Singapore 117456, Singapore

**Keywords:** single-cell RNA-seq, yeast aging, cell-to-cell heterogeneity, iron transport, mitochondrial dysfunction

Single-cell RNA-seq has been widely used to address important biological questions in higher organisms but not in microorganisms, such as the beautiful unicellular aging model of budding yeast *Saccharomyces cerevisiae* (*S*. *cerevisiae*). This is mainly due to the rigid cell wall which prevents effective cell lysis, the much smaller amount of starting RNA material and lack of polyadenylated tails on mRNAs in single yeast cells [1]. Yeast replicative aging assesses the potential of cell divisions one cell can complete, and the number of daughter cells that one mother cell produces before ceasing budding is defined as the replicative lifespan of yeast. A dilemma of replicative aging research in yeast exists for a long time between the very small proportion of old cells in the exponentially growing population and the large number of pure old cells required for conventional biochemical, genetic and transcriptomic analysis. Although several approaches including magnetic sorting, elutriation and genetic programming, have been developed to enrich old yeast cells [2, 3], it’s not yet feasible at simultaneously ensuring both the quantity and purity of the isolated old and live yeast cells. In addition, single yeast cells from isogenic populations have different replicative lifespans ultimately, and the cell-to-cell heterogeneity during aging in yeast would be of interest but may be masked by conventional bulk population analysis due to the average effect.

**Figure 1:**
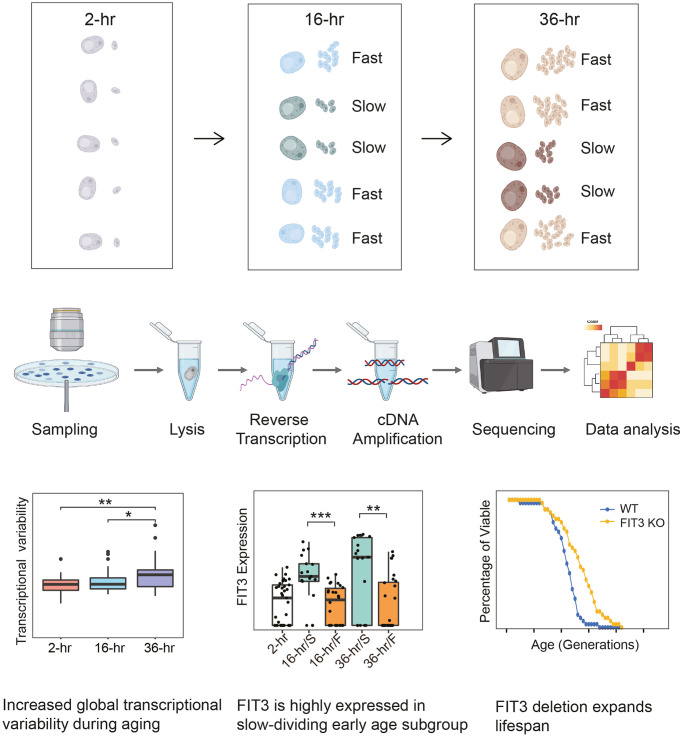
**Investigating the biology of yeast aging by single-cell RNA-seq.** We optimized a single-cell RNA-seq protocol combined with manual dissection, and performed the first transcriptome analysis of aging in yeast with single-cell resolution. By single-cell RNA-seq, we found an increased cell-to-cell transcriptional variability during aging in yeast. And we also found a molecular marker that indicates the early heterogeneity during aging in yeast: the iron transporter gene of *FIT3*. *FIT3* was reported to be induced upon iron deprivation or mitochondrial DNA loss, and in our study it was highly expressed in the slow-dividing early age subgroup. Interestingly, its deletion was proved to expand the lifespan as well.

To overcome the technical difficulties above and quantitatively investigate the biology of yeast aging with single-cell resolution, we recently optimized a single-cell RNA-seq protocol and reported the first single-cell transcriptome analysis of aging yeast [4]. We started from an observation that early yeast populations can be split into sub-populations that divide rapidly and slowly respectively in later life, as evidenced by single-cell imaging analysis in the previous study [5]. To understand the mechanisms of this early heterogeneity during replicative aging in yeast, we further manually isolated single yeast aging cells from the agar plate at three different time points: 2 hr (young), 16 hr (early age) and 36 hr (late age) after birth, and then performed single-cell RNA-seq with optimized protocol (Figure 1). Because these single yeast aging cells were obtained by manual dissection, the division rate of each cell was known. Totally 136 single yeast cells were collected with precise age for RNA-seq and our method detected on average 2,202 genes for each cell, accounting for about one third of the coding genes in budding yeast *S. cerevisiae*. The capture efficiency was more than 90% for transcripts with an absolute number of mRNA molecules above 10.

Following state-of-the-art single-cell transcriptome analysis, our study then revealed an increased cell-to-cell transcriptional variability during aging in yeast. And several key biological processes or cellular components, including oxidation-reduction process, oxidative stress response (OSR), translation, ribosome biogenesis and mitochondrion, were also highlighted that underlie aging in yeast by our global differential gene expression analysis. Indeed, the average normalized gene expression levels across age groups demonstrated an age-dependent increase in oxidation-reduction, OSR and mitochondrion, and a decrease in translation as well as ribosome biogenesis. These results were consistent with previous bulk population analysis [6].

Differential gene expression analysis of the single-cell RNA-seq data was also performed between slow- and fast-dividing age subgroups. Having done this, we uncovered a molecular marker of *FIT3* that indicates the early heterogeneity during aging in yeast. *FIT3* encodes a cell wall mannoprotein and it’s a facilitator of iron transport in yeast. Previously it was reported to be induced upon iron deprivation or mitochondrial DNA loss [7]. In our study, *FIT3* was found to be highly expressed in the slow-dividing age subgroups, and the gene expressions of *FIT3* together with several other iron transporter genes, such as *FIT2* and *FET3,* were negatively correlated with the generation of single yeast cells across different age groups; moreover, *FIT3* deletion was proved to be long-lived. Interestingly, we also found that while a large number of 145 mitochondrial genes were globally upregulated during aging in yeast, a small number of 11 different mitochondrial genes were expressed at lower levels in slow-dividing age subgroups compared to their fast-dividing counterparts with statistical significance. Among these 11 weakly expressed mitochondrial genes including *COR1*, which is a core subunit of ubiquinol-cytochrome c reductase composing complexes III, and *COX4*, which is an important component of cytochrome c oxidase belonging to complexes IV of the mitochondrial inner membrane electron transport chain. Collectively, these results suggested a relatively poor mitochondrial function in the slow-dividing cells of both early and late age subgroups. And by single-cell RNA-seq, we successfully identified a new molecular marker of *FIT3* during early aging in yeast and characterized the divergent mitochondrial gene expression profiles between age groups and subgroups that would be otherwise masked in the bulk population analysis. The regulatory variation of transcription factors during aging in yeast was also characterized at the single-cell level. *YAP1*, which is a key transcription factor responding to oxidative stress, was found to be temporally and highly activated in the early age and fast-dividing subgroup. Whereas *RPN4*, which is the transcription factor essential for proteasome biogenesis and replicative lifespan extension [8], was only prominently activated in the late age and fast-dividing subgroup.

Undoubtedly, exploiting single-cell RNA-seq enabled us to extract far more information for deciphering the molecular mechanisms of aging in yeast and other organisms. In future, the modern single-cell technologies combined with other novel methods such as nanopore sequencing with ultra-long reads are highly valuable for us to probe more deeply into the ancient problem of the biology of aging. And the mechanistic insights obtained from these efforts will provide new venues for promoting healthy aging in humans ultimately.
